# Supplementary material for: Functionally analogous body- and animacy-responsive areas are present in the dog (Canis familiaris) and human occipito-temporal lobe
Source: Commun Biol. 2023 Jun 27;6:645. doi: 10.1038/s42003-023-05014-7 (PMC10300132; doi:10.1038/s42003-023-05014-7)
Supplement: Supplementary file 2 — Supplemental Material [file 42003_2023_5014_MOESM2_ESM.pdf]

Supplementary Information

Functionally analogous body- and animacy-responsive areas are present in the dog (*Canis familiaris*) and human occipito-temporal lobe

Magdalena Boch[1,2*], Isabella C. Wagner[1], Sabrina Karl[3], Ludwig Huber[3+] & Claus Lamm[1,4+]

[1]Social, Cognitive and Affective Neuroscience (SCAN) Unit, Department of Cognition, Emotion, and Methods in Psychology, Faculty of Psychology, University of Vienna, Vienna, Austria, [2]Department of Cognitive Biology, Faculty of Life Sciences, University of Vienna, Vienna, Austria, [3]Comparative Cognition, Messerli Research Institute, University of Veterinary Medicine Vienna, Medical University of Vienna and University of Vienna, Vienna, Austria, [4]Vienna Cognitive Science Hub, University of Vienna, Vienna, Austria

*Corresponding author: magdalena.boch@univie.ac.at

[+] These authors contributed equally.

Validation of threshold to define individual functional regions-of-interest

Supplementary Note 1

Individual functional regions-of-interest (fROIs) were determined based on the top-10% most active voxels within each anatomical search space. To validate the chosen threshold and investigate, for example, if smaller fROI sizes would have been able to detect face-sensitive areas in the dog brain, we extracted parameter estimates for percentage cut-offs ranging from 1% to 100%. The results show that smaller fROI sizes were less sensitive in all anatomical search spaces in the dog brain (see **Supplementary Figure S1**). In the human fusiform face area and occipital face area, thresholds above 60% resulted in fROIs that could not detect differences in activation levels between faces and bodies. Thus, the fROIs likely also contained voxels that are not face- or body-sensitive at this size. For the dogs, larger fROI sizes revealed comparable outcomes in the mid and caudal suprasylvian gyrus but had less sensitivity in the ectomarginal gyrus than lower thresholds. Thus, comparing fROI-defining thresholds confirmed 10% as a sufficient threshold to define category-sensitive fROIs in the dog and human brain.

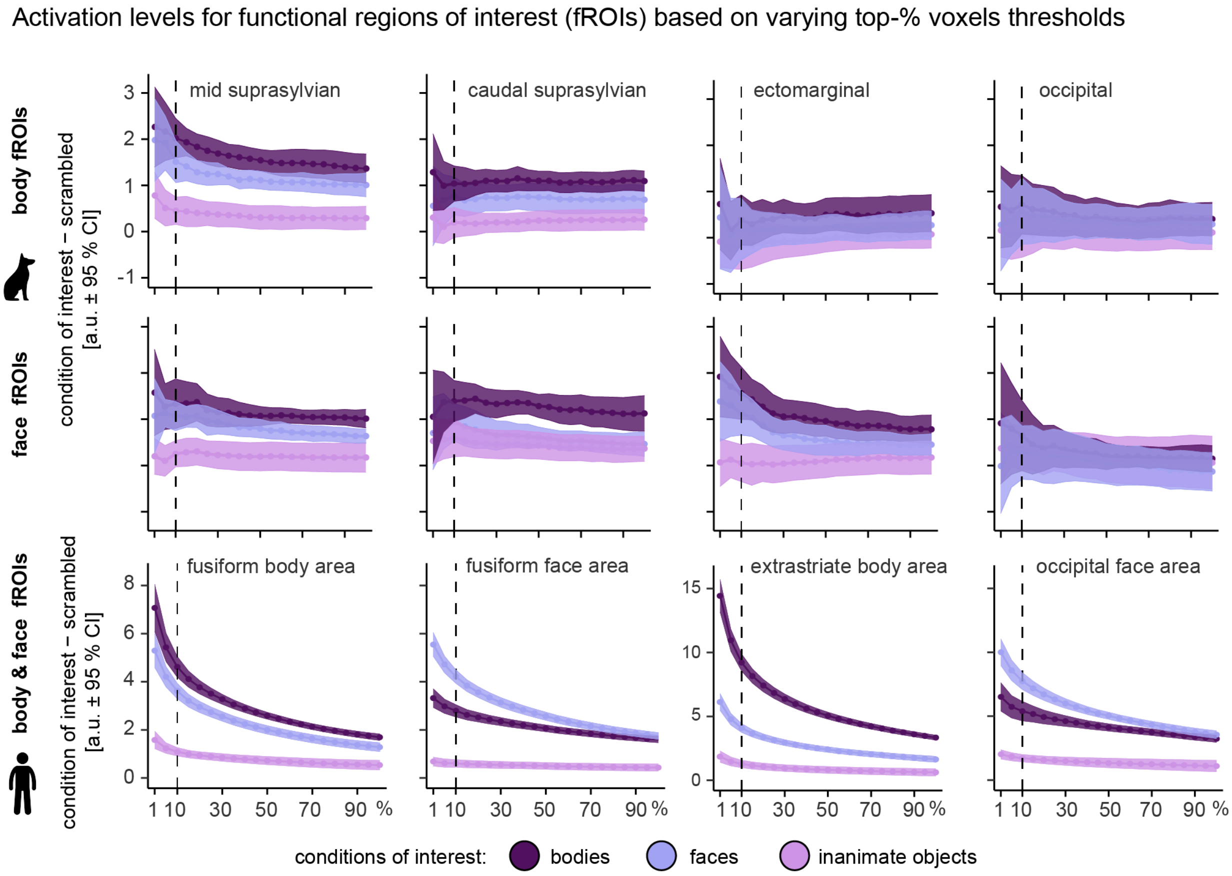

Supplementary Figure S1. *Exploratory analysis of parameter estimates for faces, bodies, and inanimate objects (all contrasts against scrambled controls) retrieved from individual functional regions of interest (fROIs) defined based on top-% most active voxels for faces or bodies > inanimate objects (run 1) ranging from 1% to 100% in steps of 5%.* For dogs, fROIs defined based on thresholds below 10% were less sensitive in all anatomical search spaces, and thresholds above 60% were less sensitive in the human fusiform and occipital face area, as illustrated by the overlapping 95% confidence intervals (CIs). Points represent the mean. a.u., arbitrary units. The dashed line represents the a priori selected 10% threshold used for the main analysis we set out to validate. Conditions of interest: bodies (purple), faces (lilac), inanimate objects (pink). The dog and human icons in panel **a** were purchased from thenounproject.com (royalty-free license).

Control analysis matching the analysis approach used for the dog neuroimaging data

Supplementary Note 2

Since anatomical search space definitions and sample sizes differed between the two species, we conducted a control analysis with the human data using anatomical masks instead of parcels (i.e., matching the analysis in the dogs). We performed the analysis in 1000 randomly drawn sub-samples of n = 15 participants (i.e., the dog sample size). We selected the calcarine sulcus, including the surrounding cortex, and the fusiform gyrus as anatomical masks to test both primary and higher-order visual regions[1], similar to the dog analysis. Analysis with the total sample of N = 40 human participants revealed no difference in activation levels between faces, bodies, and inanimate objects in the calcarine body fROI and negative activation levels for all three categories compared to baseline (i.e., scrambled control condition) in the calcarine face fROI; differences between faces and bodies in the calcarine fROIs were both non-significant in more than 5% of the sub-samples (see **Supplementary Figures S2-S3**). Results for the fusiform face fROI revealed greater activation levels for faces compared to bodies and inanimate objects in the total sample and a significant difference between faces and bodies in 100% of the sub-sample analyses. The fusiform body fROI also showed greater activation levels for bodies compared to faces and inanimate objects in the total sample and 97.5% of the sub-samples. Thus, the observed differences between dogs and humans (i.e., face-sensitivity) are also present when accounting for sample size and search space differences (see **Supplementary Figures S2-S3**).

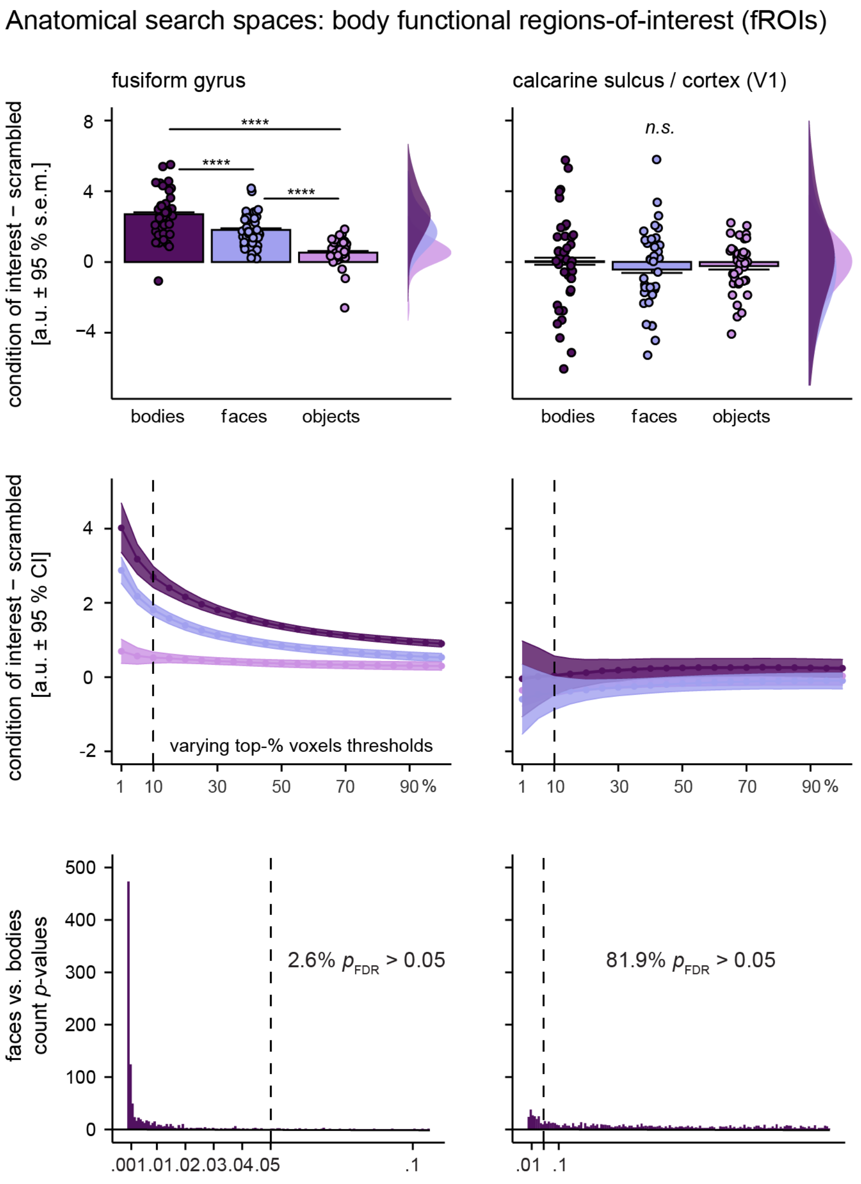

Supplementary Figure S2. *Identical functional regions-of-interest (fROI) analysis approach as in dogs revealing a body-sensitive region in the human fusiform gyrus but no category-sensitivity in the primary visual cortex*. Using anatomical masks instead of parcels to define individual body fROIs revealed evidence for body-sensitivity in the human fusiform gyrus (F(1.90,74.25) = 115.31,p_{FDR} < .0001, η^2_g = .443) but not the calcarine cortex (F(1.41,77.79) = 1.43, p_{FDR} < .245, η^2_g = .008) in the full sample of N = 40 human participants (first row). Second row: Visual inspection of parameter estimates for faces, bodies and inanimate objects (all > scrambled images) confirms top-10% active voxels as sufficient fROI defining threshold for all fROIs. Running the analysis with 1000 randomly drawn sub-samples of n = 15 human participants (i.e., equivalent to dog sample size) confirmed again greater activation for bodies compared to faces in 97.4% of the cases (i.e., less than 5% nonsignificant comparisons) in the fusiform body fROI and no significant difference between bodies and faces in calcarine fROI in 81.9% of the cases. Planned comparisons were false discovery rate (FDR) corrected to control for multiple comparisons. ****p_{FDR} < .0001, error bars represent the standard error of the mean (s.e.m; left panel) or the 95% confidence interval (CI) of the mean in the middle panel; n.s., not significant; a.u., arbitrary units; V1, primary visual cortex. Conditions of interest: bodies (purple), faces (lilac), inanimate objects (pink).

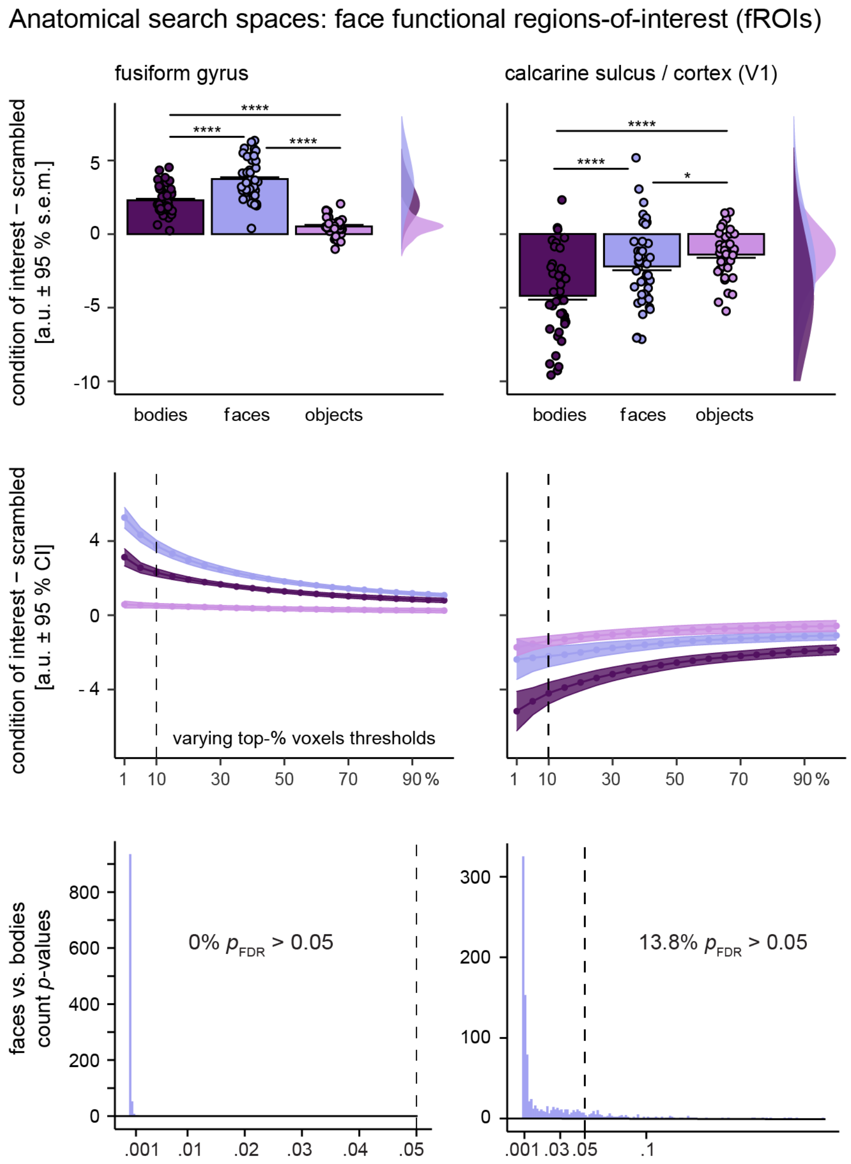

Supplementary Figure S3. *Identical functional regions-of-interest (fROI) analysis approach as in dogs revealing a face-sensitive region in the human fusiform gyrus but not in the primary visual cortex*. Using anatomical masks instead of parcels to define individual face fROIs, revealed evidence for face-sensitivity in the human fusiform gyrus ($F(1.82,70.86) = 214.45$, $p_{FDR} < .0001$, $\eta^2_g = .622$) in the full sample of $N = 40$ human participants (first row). In the calcarine cortex, activation levels between faces and bodies were significantly different ($F(1.94,75.68) = 34.01$, $p_{FDR} < .0001$, $\eta^2_g = .187$) but activation levels for faces, bodies, and inanimate objects were negative indicating less for all categories compared to scrambled images. Second row: Visual inspection of parameter estimates for faces, bodies, and inanimate objects (all > scrambled images) confirms top-10% active voxels as sufficient fROI defining threshold for all fROIs. Running the analysis with 1000 randomly drawn sub-samples of $n = 15$ human participants (i.e., equivalent to dog sample size) confirmed again greater activation for faces compared to bodies in 100% of the cases in the fusiform face fROI. In the calcarine cortex differences between faces and bodies were not significant in 13.8%. Planned comparisons were false discovery rate (FDR) corrected to control for multiple comparisons. *$p_{FDR} < .05$, ****$p_{FDR} < .0001$, error bars represent the standard error of the mean (left panel) or the 95% confidence interval (CI) of the mean (s.e.m.) in the middle panel; a.u., arbitrary units; V1, primary visual cortex. Conditions of interest: bodies (purple), faces (lilac), inanimate objects (pink).

Exploratory analysis of low-level visual properties of the stimulus set

Supplementary Note 3

To control for low-level visual differences, the stimulus set was controlled for size, spatial extent, and luminance (see **Stimulus Material** for detailed information). We did not control for other low-level visual properties to preserve ecological validity (i.e., dog fur and human clothing do differ in hue in everyday life). However, to test if low-level visual properties might have driven the observed effects, we conducted exploratory analyses comparing hue, saturation, and contrast measures between face, body, and inanimate object stimuli and between dog and human face and body stimuli (i.e., mirroring the fROI analysis). Results revealed no differences in contrast between stimuli categories but expected differences in hue and saturation (see **Figure S4**, **Supplementary Tables S7-S9**): hue differed between bodies and inanimate objects as well as between faces and inanimate objects, but not between faces and bodies. Comparing dog and human stimuli, we found a significant difference in hue between dog and human bodies. Faces and inanimate objects did not differ in saturation, but body stimuli had a lower saturation compared to faces and inanimate objects, and dog faces had a lower saturation compared to human faces. These findings indicate that the observed differences in the dog and human fROI analysis do not reflect low-level visual differences. For example, if dog fROIs were driven by differences in hue and saturation, we should have also seen differences in activation levels in response to human and dog bodies (i.e., hue) or dog and human faces (i.e., saturation).

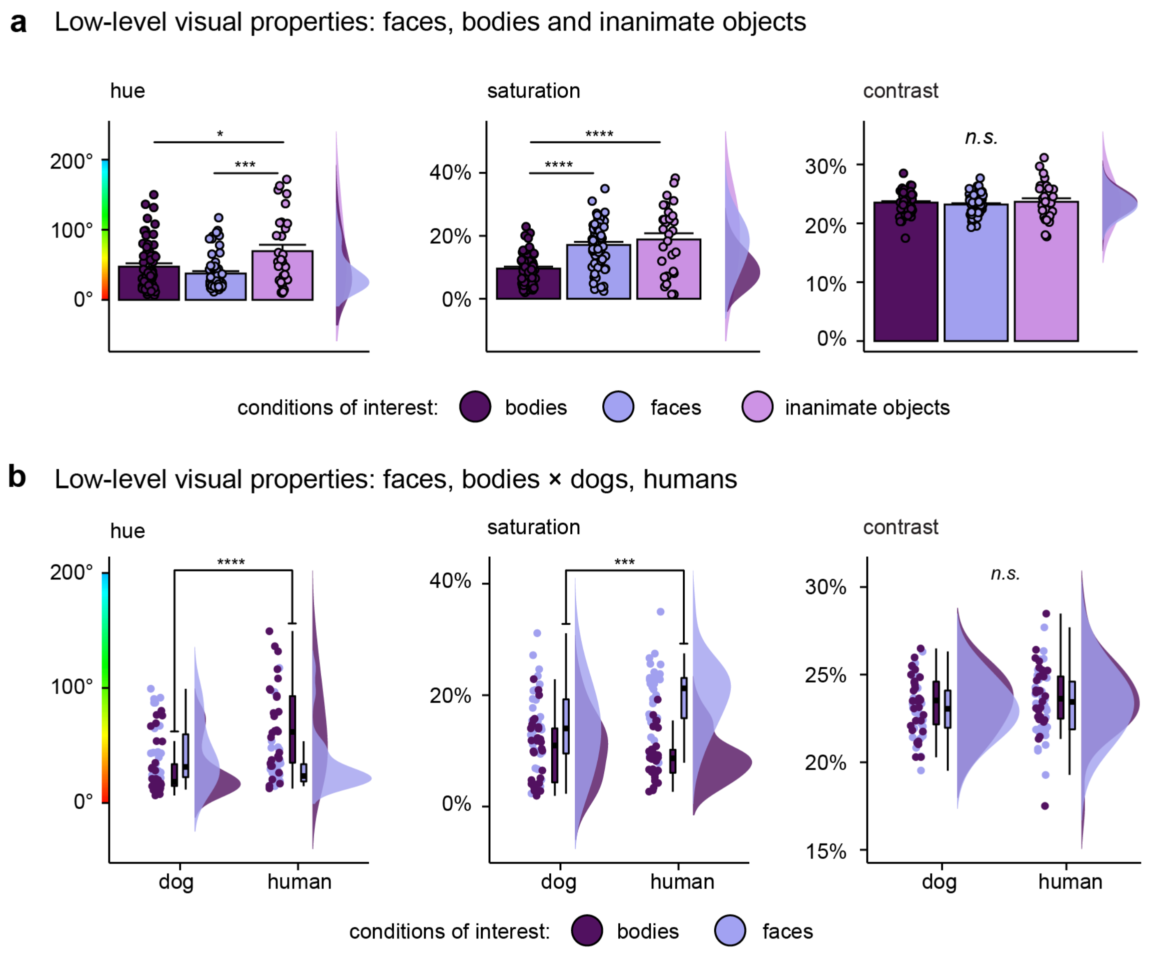

Supplementary Figure S4. *Exploratory analysis shows low-level visual differences between conditions for hue and saturation, but not for contrast.* Hue represents degrees (°) on a colour wheel (range: 0° to 360°), and saturation and contrast are measured in percentages (range: 0% to 100%). (**a**) Hue of face and body stimuli each differ from inanimate objects, (**b**) adding species as factor further showed a difference between dog and human body stimuli. (**a**) Body stimuli have significantly lower saturation than face or inanimate object stimuli. The latter do not differ in saturation, (**b**) but within the face stimuli, saturation levels are significantly higher for human compared to dog faces. (**a**, **b**) We did not find any differences between stimuli categories for contrast. Detailed information can be found in **Supplementary Tables S7-S8**. The stimulus set has been controlled for luminance, spatial extent (= ratio image / background) and size (see section **Stimulus material** for details). Post-hoc comparisons were false discovery rate (FDR) corrected to control for multiple comparisons *p < .05, ***p < .001, ****p < .0001. Error bars represent the standard error of the mean. Conditions of interest: bodies (purple), faces (lilac), inanimate objects (pink).

Supplementary Table S1. Task-related activation: localizer data set (task run 1)

Contrast & brain region	Coordinates			*z*-value	cluster size
	x	y	z		
**All conditions > implicit visual baseline**					
**Dog participants**					
R mid suprasylvian gyrus	-16	-22	14	4.56	416
L caudal suprasylvian gyrus	-22	-26	0	4.39	44
R caudal suprasylvian gyrus	22	-24	0	4.04	45
**Human participants**					
R middle occipital gyrus	48	-70	-8	Inf	40800
R middle frontal gyrus	54	12	40	5.41	1360
L middle temporal gyrus	-64	-8	-16	5.31	73
L superior frontal gyrus	-4	14	56	4.72	128
R superior frontal gyrus	4	14	-12	4.69	148
L superior frontal gyrus	-10	62	48	4.57	78

Note. Effects were tested for significance with a cluster defining threshold of p <.005/.001 (dogs/humans) and a cluster probability threshold of $p < .05$ FWE corrected for multiple comparisons. We report the first local maximum within each cluster of each sample *t*-test. Coordinates for the dog data represent the location of the peak voxels referring to the canine breed-averaged template space[2]; the template along with another detailed dog anatomical atlas[3] served to determine anatomical nomenclature for the dog data. Human data coordinates refer to the stereotactic Montreal Neurological Institute (MNI) space, we obtained anatomical nomenclature from the Laboratory for Neuro Imaging (LONI) Brain Atlas[4], LBPA40, http://www.loni.usc.edu/atlases/). Data of both samples is presented in **Figure 1c**. L, left; R, right.

Supplementary Table S2. fROI sizes with top-10% active voxels cut-off

	left		right	
Sample, search space and fROI	mean	*SD*	mean	*SD*
**Dog participants: body fROIs**				
Mid suprasylvian gyrus	8.33	2.97	11.13	3.7
Caudal suprasylvian gyrus	10.73	2.76	10	4.49
Ectomarginal gyrus	9.47	4.32	9.2	3.76
Marginal gyrus	8.93	3.45	8.87	2.77
Splenial gyrus	12.73	5.35	8.8	4.23
Occipital gyrus	4.6	2.03	5.52	3.09
**Dog participants: face fROIs**				
Mid suprasylvian gyrus	10.6	3.36	9.07	3.61
Caudal suprasylvian gyrus	8.4	2.87	7.2	3.34
Ectomarginal gyrus	11.47	4.73	9.07	3.56
Marginal gyrus	9.8	4.3	12.33	3.37
Splenial gyrus	14.27	5.18	12.4	4.64
Occipital gyrus	5.93	2.58	7.73	2.63
**Human participants: body fROIs**				
Extrastriate body area	168.25	30.04	204.8	29.09
Fusiform body area	70.85	29.24	84	25.4
Anatomical mask: fusiform body area	71.62	27.09	79.35	28.66
**Human participants: face fROIs**				
Occipital face area	14.72	15.83	48.5	15.97
Fusiform face area	28.73	10.92	64	16.09
Anatomical mask: fusiform face area	69	30.82	72.17	30.95

Note. Summary statistics for individual face and body functional regions-of-interest (fROIs) split for right and left hemisphere.*SD*, standard deviation.

Supplementary Table S3. Results from one-way[a] repeated measures analyses of variance (ANOVAs)

Search space (predictor: image category)	df_{Num}	df_{Den}	F	p	p_{FDR}	η^2_g
**Dog participants**						
**Individual body fROIs**						
Mid suprasylvian gyrus	1.77	24.78	23.83	**< .0001**	**< .0001**	.63
Caudal suprasylvian gyrus	1.60	22.47	7.16	**.006**	**.018**	.34
Ectomarginal gyrus	2	27.99	1.23	.308	.37	.081
Marginal gyrus	1.85	25.96	1.19	.317	.317	.078
Splenial gyrus	1.98	27.79	2.06	.146	.292	.129
Occipital gyrus	1.89	26.45	1.56	.230	.345	.10
**Individual face fROIs**						
Mid suprasylvian gyrus	1.77	24.75	8.19	**.003**	**.009**	.369
Caudal suprasylvian gyrus	1.93	26.96	4.56	**.021**	**.042**	.246
Ectomarginal gyrus	1.77	24.81	9.41	**.001**	**.006**	.402
Marginal gyrus	1.85	25.87	2.60	.097	.146	.157
Splenial gyrus	1.42	19.82	.57	.518	.622	.039
Occipital gyrus	1.97	27.52	.40	.671	.671	.028
**Human participants**						
**Individual body fROIs**						
Extrastriate body area	1.44	56.19	264	**< .0001**	**< .0001**	.871
Fusiform body area	1.92	75.02	129.9	**< .0001**	**< .0001**	.769
**Individual face fROIs**						
Occipital face area	1.87	72.77	108.63	**< .0001**	**< .0001**	.736
Fusiform face area	1.94	75.85	206.35	**< .0001**	**< .0001**	.841

Note. [a]Independent variable: *image category* (levels: bodies, faces, inanimate objects; all over scrambled control), dependent variable: activation levels. For each sample (i.e., dogs and humans) *p*-values for group comparisons investigating the same research question (i.e., body-preference for bodies) are false discovery rate (FDR) corrected, uncorrected *p*-values are also reported. *P*-values < .05 are in bold. Post-hoc comparison results are presented in **Figure 2b**. df_{Num}, degrees of freedom numerator; df_{Den} degrees of freedom denominator; η^2_g, generalized eta-squared.

Supplementary Table S4. Whole-brain univariate analysis: face-, body- and animacy-sensitivity (dog data)

Contrast & brain region	Coordinates			*z*-value	cluster size
	x	y	z		
**Bodies > faces, inanimate objects**					
L mid suprasylvian gyrus	-16	-22	13	5.63	67
R caudal suprasylvian gyrus	20	-26	4	4.48	129
L caudal suprasylvian gyrus	-20	-24	2	4.33	42
**Animate (faces, bodies) > inanimate objects**					
L mid suprasylvian gyrus	-18	-24	16	5.35	60

Note. Effects were tested for significance with a cluster defining threshold of p <.005 and a cluster probability threshold of p < .05 FWE corrected for multiple comparisons. We report the first local maximum within each cluster for each post hoc sample t-tests, the critical cluster size to determine significance was k = 39. Post-hoc comparisons for faces > bodies, inanimate objects did not survive multiple comparison corrections. Coordinates represent the location of the peak voxels referring to the canine breed-averaged template space[2]; the template along with another detailed dog atlas[3] served to determine anatomical nomenclature. The corresponding activation maps are visualized in **Figure 3b**. L, left; R, right.

Supplementary Table S5. Whole-brain univariate analysis: face-, body- and animacy-sensitivity (human data)

Contrast & brain region	Coordinates			*z*-value	cluster size
	x	y	z		
**Bodies > faces, inanimate objects**					
L middle occipital gyrus	-46	-70	10	Inf	20866
R middle frontal gyrus	30	-2	50	6.16	1303
R lingual gyrus	22	-64	-4	6.05	1050
L thalamus	-18	-30	4	6.03	87
L middle frontal gyrus	-28	-6	52	5.9	979
**Faces > bodies, inanimate objects**					
R fusiform gyrus	42	-50	-22	7.8	999
R hippocampus	18	-4	-16	7.08	864
L inferior occipital gyrus	-38	-88	-14	6.90	454
L hippocampus	-18	-4	-16	6.44	638
R precuneus	4	-54	32	5.99	741
**Animate (faces, bodies) > inanimate objects**					
R middle occipital gyrus	46	-70	-4	Inf	5144
L middle occipital gyrus	-48	-80	-2	Inf	4554
R hippocampus	18	-4	-14	6.96	896
L hippocampus	-18	-6	-16	6.35	651
R superior parietal gyrus	30	-48	56	6.12	4189

Note. Effects were tested for significance with a cluster defining threshold of $p < .001$ and a cluster probability threshold of $p < .05$ FWE corrected for multiple comparisons. We report the first local maximum within each cluster for each post hoc sample *t*-tests limited to the first five cluster with the highest peak *z*-values; the critical cluster size to determine significance was $k = 66$ voxels. Coordinates represent the location of the peak voxels referring to the stereotactic Montreal Neurological Institute (MNI) space. We obtained anatomical nomenclature from the Laboratory for Neuro Imaging (LONI) Brain Atlas[4], LBPA40, http://www.loni.usc.edu/atlases/) and the Automatic Anatomical Labelling atlas (AAL2; Rolls, Joliot and Tzourio-Mazoyer, 2015). Data is presented in **Figure 3b**. L, left; R, right.

Supplementary Table S6. Results from 2 × 2 repeated measures analyses of variance (ANOVAs) [a]

Search space, predictor	df_{Num}	df_{Den}	F	*p*	p_{FDR}	η^2_g
**Dog participants**						
**Individual body fROIs**						
Mid suprasylvian gyrus						
Species	1	14	1.40	.257	.514	.091
Animate stimulus	1	14	7.19	**.018**	.108	.339
Species × animate stimulus	1	14	1.37	.261	.392	.089
Caudal suprasylvian gyrus						
Species	1	14	4.70	**.048**	.144	.251
Animate stimulus	1	14	1.08	.317	.380	.071
Species × animate stimulus	1	14	.03	.855	.855	.002
**Individual face fROIs**						
Mid suprasylvian gyrus						
Species	1	14	.99	.337	.433	.066
Animate stimulus	1	14	1.06	.32	.48	.071
Species × animate stimulus	1	14	1.81	.2	.36	.114
Caudal suprasylvian gyrus						
Species	1	14	0	.995	.995	< .001
Animate stimulus	1	14	2.95	.108	.324	.174
Species × animate stimulus	1	14	.41	.535	.602	.028
Ectomarginal gyrus						
Species	1	14	3.55	.08	.72	.202
Animate stimulus	1	14	1.83	.198	.446	.116
Species × animate stimulus	1	14	3.25	.093	.419	.188
**Human participants**						
**Individual body fROIs**						
Extrastriate body area						
Species	1	39	3.29	.077	.77	.078
Animate stimulus	1	39	219.45	**< .0001**	**< .0001**	.849
Species × animate stimulus	1	39	15.33	**< .0001**	**< .0001**	.282
Fusiform body area						
Species	1	39	.31	.579	.695	.008
Animate stimulus	1	39	63.43	**< .0001**	**< .0001**	.312
Species × animate stimulus	1	39	4.41	**.042**	.063	.044
**Individual face fROIs**						
Occipital face area						
Species	1	39	.01	.917	.917	< .001
Animate stimulus	1	39	69.57	**< .0001**	**< .0001**	.641
Species × animate stimulus	1	39	1.36	.251	.301	.034
Fusiform face area						
Species	1	39	15.77	**< .001**	**< .001**	.288
Animate stimulus	1	39	102.16	**< .0001**	**< .0001**	.724
Species × animate stimulus	1	39	1.44	.237	.356	.036

Note. [a]Independent variables are *species* (levels: dog, human) and *animate stimulus* (levels: bodies, faces), all over scrambled control; dependent variable: activation levels. For each participant group, *p*-values for group comparisons investigating the same research question (i.e., species-preference) are FDR (false discovery rate) corrected, uncorrected *p*-values are also reported. *P*-values < .05 are in bold. Post-hoc comparison results are presented in **Figure 4**. df_{Num}, degrees of freedom numerator; df_{Den} degrees of freedom denominator; η^2_g, generalized eta-squared.

Supplementary Table S7. Results from one-way analyses of variance (ANOVAs)[a]

Visual property (predictor: image category)	df_{Num}	df_{Den}	F	*p*	η^2_g
**Hue**	2	147	7.94	**< .001**	.098
**Saturation**	2	147	22.28	**< .001**	.233
**Contrast**	2	147	.58	.560	.008

Note. [a]Independent variable: *image category* (levels: bodies, faces, inanimate objects), dependent variable: measurement of low-level visual property (i.e., hue, saturation, contrast). *P*-values < .05 are displayed in bold. Post-hoc comparison results are presented in **Figure S4a**. df_{Num}, degrees of freedom numerator; df_{Den} degrees of freedom denominator; η^2_g, generalized eta-squared.

Supplementary Table S8. Results from two-way analyses of variance (ANOVAs)[a]

Measurement, predictor	df_{Num}	df_{Den}	F	*p*	η^2_g
**Hue**					
Species	1	116	5.45	**.021**	.045
Animate stimulus	1	116	3.56	.062	.03
Species × animate stimulus	1	116	20.24	**<.001**	.149
**Saturation**					
Species	1	14	4.39	**.038**	.036
Animate stimulus	1	14	48.36	**<.001**	.294
Species × animate stimulus	1	14	10.87	**<.001**	.086
**Contrast**					
Species	1	14	.76	.329	.007
Animate stimulus	1	14	.96	.384	.008
Species × animate stimulus	1	14	0	.950	<.001

Note. [a]Independent variables: *animate stimulus* (levels: bodies, faces), *species* (levels: dogs, humans), dependent variable: measurement of low-level visual property (i.e., hue, saturation, contrast). *P*-values < .05 are displayed in bold. Post-hoc comparison results are presented in **Figure S4b.** df_{Num}, degrees of freedom numerator; df_{Den} degrees of freedom denominator; η^2_g, generalized eta-squared.

Supplementary Table S9. Dog data: Pattern similarity during face, body, and object perception

Contrast & brain region	Coordinates			t-value	cluster size
	x	y	z		
Bodies [dog bodies × human bodies] > inanimate objects					
R caudal suprasylvian gyrus	18	-24	4	5.77	116
Faces [dog faces × human faces] > inanimate objects					
R marginal gyrus	5	-34	20	7.01	245
R olfactory tuberculum	8	8	-2	6.16	80
Conspecific (dog) bodies > heterospecific (human) bodies					
R mid ectosylvian gyrus	16	-16	12	5.53	62
L caudal suprasylvian gyrus	-16	-27	4	5.21	99
L piriform lobe	-14	-3	-4	5.18	95
Animate [dog bodies × human bodies × dog faces × human faces] > inanimate stimuli					
R splenial gyrus	5	-32	7	5.38	70
R caudal suprasylvian gyrus	18	-24	2	5.25	72

Note. Effects were tested for significance with a cluster defining threshold of p <.005 and a cluster probability threshold of p < .05 FWE corrected for multiple comparisons. We report the first local maximum within each cluster for all paired sample t-tests. The results from the paired sample t-tests comparing faces [dog faces × human faces] vs. bodies [dog bodies × human bodies] and conspecific (dog) faces vs. heterospecific (human) faces along with the reversed contrasts [dog bodies × human bodies] < inanimate objects, [dog faces × human faces] < inanimate objects, conspecific (dog) bodies < heterospecific (human) bodies and [dog bodies × human bodies × dog faces × human faces] < inanimate objects did not survive the statistical threshold. Coordinates represent the location of the peak voxels referring to the canine breed-averaged template space[2]; the template along with another dog atlas[3] served to determine anatomical nomenclature for the dog data. Data is presented in **Figure 5**. L, left; R, right.

Supplementary Table S10. Human data: Pattern similarity during face, body, and object perception

Contrast & brain region	Coordinates			*t*-value	cluster size
	x	y	z		
Bodies [dog bodies × human bodies] > inanimate objects					
R middle occipital gyrus	56	-72	6	14.98	14018
L middle occipital gyrus	-54	-78	10	12.32	1622
L cerebellum	-4	-70	-40	7.33	585
L superior parietal gyrus	-26	-62	68	6.65	1383
R superior frontal gyrus	6	62	-4	6.17	881
L precentral gyrus	-26	-10	50	5.26	570
Bodies [dog bodies × human bodies] < inanimate objects					
L inferior temporal gyrus	-22	-48	-12	11.26	6678
Faces [dog faces × human faces] > inanimate objects					
R fusiform gyrus	46	-42	-20	9.35	1088
R middle occipital gyrus	56	-72	0	5-71	349
Faces [dog faces × human faces] < inanimate objects					
L cerebellum	-20	-54	-18	14.20	7002
R lingual gyrus	24	-56	-8	11.84	5198
L precentral gyrus	-46	6	32	4.77	353
Faces [dog faces × human faces] > bodies [dog bodies × human bodies]					
R lingual gyrus	6	-88	0	11.35	3461
Faces [dog faces × human faces] < bodies [dog bodies × human bodies]					
R middle occipital gyrus	34	-72	10	14.21	39057
R middle orbitofrontal gyrus	24	42	-14	5.53	1087
R cerebellum	8	-72	-38	5.19	572
R cingulate gyrus	2	6	26	4.76	267
Conspecific (human) bodies > heterospecific (dog) bodies					
R fusiform gyrus	44	-44	-22	5.76	1053
L middle occipital gyrus	-54	-72	12	4.87	267
R middle temporal gyrus	40	-54	12	4.81	468
R superior parietal gyrus	16	-54	42	4.60	466
Heterospecific (dog) faces > conspecific (human) faces					
L middle occipital gyrus	-16	-94	6	7.20	8147
Brainstem	6	-40	-26	4.64	618
Animate [dog bodies × human bodies × dog faces × human faces] > inanimate objects					
R middle occipital gyrus	56	-72	0	9.09	2035
L cerebellum	-4	-70	-42	7.30	277
R postcentral gyrus	36	-26	48	5.09	403
R superior parietal gyrus	38	-44	68	4.82	338
R superior frontal gyrus	8	52	-8	4.77	564
Animate [dog bodies × human bodies × dog faces × human faces] < inanimate objects					
L cerebellum	-22	-50	-18	12.63	11878

Note. Effects were tested for significance with a cluster defining threshold of p <.001 and a cluster probability threshold of p < .05 FWE corrected for multiple comparisons. We report the first local maximum within each cluster for all paired sample *t*-tests. Results from the reversed contrasts conspecific (human) bodies < heterospecific (dog) bodies and heterospecific (dog) faces < conspecific (human) faces did not survive the statistical threshold. Coordinates represent the location of the peak voxels and we obtained anatomical nomenclature from the Laboratory for Neuro Imaging (LONI) Brain Atlas[4], LBPA40, http://www.loni.usc.edu/atlases/). Data is presented in **Figure 5.**

Supplementary References

1. Grill-Spector, K. & Malach, R. The Human Visual Cortex. *Annu. Rev. Neurosci.* **27**, 649–677 (2004).
2. Nitzsche, B. *et al.* A stereotaxic breed-averaged, symmetric T2w canine brain atlas including detailed morphological and volumetrical data sets. *Neuroimage* **187**, 93–103 (2019).
3. Czeibert, K., Andics, A., Petneházy, Ö. & Kubinyi, E. A detailed canine brain label map for neuroimaging analysis. *Biol. Futur.* **70**, 112–120 (2019).
4. Shattuck, D. W. *et al.* Construction of a 3D probabilistic atlas of human cortical structures. *Neuroimage* **39**, 1064–1080 (2008).
5. Rolls, E. T., Joliot, M. & Tzourio-Mazoyer, N. Implementation of a new parcellation of the orbitofrontal cortex in the automated anatomical labeling atlas. *Neuroimage* **122**, 1–5 (2015).
