# Supplementary material for: Functionally analogous body- and animacy-responsive areas are present in the dog (Canis familiaris) and human occipito-temporal lobe
Source: Commun Biol. 2023 Jun 27;6:645. doi: 10.1038/s42003-023-05014-7 (PMC10300132; doi:10.1038/s42003-023-05014-7)
Supplement: Supplementary file 3 — Reporting Summary [file 42003_2023_5014_MOESM3_ESM.pdf]

nature portfolio

Corresponding author(s): Magdalena Boch

Last updated by author(s): May 24, 2023

Reporting Summary

Nature Portfolio wishes to improve the reproducibility of the work that we publish. This form provides structure for consistency and transparency in reporting. For further information on Nature Portfolio policies, see our Editorial Policies and the Editorial Policy Checklist.

Statistics

For all statistical analyses, confirm that the following items are present in the figure legend, table legend, main text, or Methods section.

n/a	Confirmed	
[ ]	[x]	The exact sample size (*n*) for each experimental group/condition, given as a discrete number and unit of measurement
[ ]	[x]	A statement on whether measurements were taken from distinct samples or whether the same sample was measured repeatedly
[ ]	[x]	The statistical test(s) used AND whether they are one- or two-sided<br>*Only common tests should be described solely by name; describe more complex techniques in the Methods section.*
[x]	[ ]	A description of all covariates tested
[ ]	[x]	A description of any assumptions or corrections, such as tests of normality and adjustment for multiple comparisons
[ ]	[x]	A full description of the statistical parameters including central tendency (e.g. means) or other basic estimates (e.g. regression coefficient) AND variation (e.g. standard deviation) or associated estimates of uncertainty (e.g. confidence intervals)
[ ]	[x]	For null hypothesis testing, the test statistic (e.g. *F*, *t*, *r*) with confidence intervals, effect sizes, degrees of freedom and *P* value noted<br>*Give P values as exact values whenever suitable.*
[x]	[ ]	For Bayesian analysis, information on the choice of priors and Markov chain Monte Carlo settings
[x]	[ ]	For hierarchical and complex designs, identification of the appropriate level for tests and full reporting of outcomes
[ ]	[x]	Estimates of effect sizes (e.g. Cohen's *d*, Pearson's *r*), indicating how they were calculated

Our web collection on statistics for biologists contains articles on many of the points above.

Software and code

Policy information about availability of computer code

Data collection	The task was implemented using the PsychoPy-Psychophysics software in Python (Peirce 2007 J. Neurosci. Methods) and neuroimaging data was collected using a Siemens Skyra 3 Tesla MRI scanner (Siemens Healthineers, Erlangen ,Germany).
Data analysis	Data was analysed using SPM12 (https://www.fil.ion.ucl.ac.uk/spm/software/spm12/), Matlab 2018b (MathWorks) and R 3.6.3. To create figures we mainly used the R packages ggplot2 (https://CRAN.R-project.org/package=ggplot2) and RainCloudPlots (Poggiali et al. 2019 Wellcome Open Res.), and the python project nilearn (http://nilearn.github.io), as well as itk-SNAP (Yushkevich et al. 2006 Neuroimage) and MRIcron (https://www.nitrc.org/projects/mricron). Custom code and data to reproduce the analysis and figures are openly available at the Open Science Framework (https://osf.io/kzcs2/) and Github (https://github.com/magdalenaboch/fROI-analysis, https://github.com/isabellawagner/searchlight-rsa).

For manuscripts utilizing custom algorithms or software that are central to the research but not yet described in published literature, software must be made available to editors and reviewers. We strongly encourage code deposition in a community repository (e.g. GitHub). See the Nature Portfolio guidelines for submitting code & software for further information.

Data

Policy information about availability of data

All manuscripts must include a data availability statement. This statement should provide the following information, where applicable:

- Accession codes, unique identifiers, or web links for publicly available datasets
- A description of any restrictions on data availability
- For clinical datasets or third party data, please ensure that the statement adheres to our policy

Univariate and multivariate beta maps, individual functional region-of-interest (fROI) data, motion parameters, low-level visual properties descriptives of the stimulus material and further sample descriptives have been deposited at the Open Science Framework (OSF) and are publicly available at https://osf.io/kzcs2/. Raw human neuroimaging data is made available upon reasonable request and raw dog neuroimaging data is publicly available at Zenodo (https://doi.org/10.5281/zenodo.7691966).

Human research participants

Policy information about studies involving human research participants and Sex and Gender in Research.

Reporting on sex and gender	We collected data from N = 40 human participants (22 females). Sex was determined based on self-reporting, we did not determine gender of the participants. Sex or gender differences were not considered in the analysis since we investigated basic cognitive mechanisms (i.e., visual perception of faces and bodies).
Population characteristics	Age of the human participants ranged between 19-28 years (mean: 23 years). Human participants were right-handed, had normal or corrected-to-normal vision, reported no history of neurological or psychiatric disease or phobia of dogs and fulfilled the standard inclusion criteria for functional MRI.
Recruitment	Human participants were recruited via the Study Participant Platform of the Cognitive Science Hub at the University of Vienna (https://cognitivescience.univie.ac.at/services/study-participant-platform/)
Ethics oversight	Human data collection was approved by the ethics committee of the University of Vienna (reference number: 00565) and performed in line with the latest revision of the Declaration of Helsinki (2013).

Note that full information on the approval of the study protocol must also be provided in the manuscript.

Field-specific reporting

Please select the one below that is the best fit for your research. If you are not sure, read the appropriate sections before making your selection.

☒ Life sciences ☐ Behavioural & social sciences ☐ Ecological, evolutionary & environmental sciences

For a reference copy of the document with all sections, see nature.com/documents/nr-reporting-summary-flat.pdf

Life sciences study design

All studies must disclose on these points even when the disclosure is negative.

Sample size	N = 15 trained family pet dogs consisting of 10 Border Collies, 2 Australian Shepherds, 1 Labrador Retriever and 2 mixed-breed dogs participated in the present study. We aimed to collect data from a minimum of N = 12 dogs during the data collection period from April 2019 to July 2020 which was the median sample size of task-based dog fMRI studies at the time of planning the study (the median sample size in 2022 was N = 13.5).<br>We collected comparative data from N = 40 human participants. We aimed for a sample size of N = 40 participants, based on previous studies in our lab with similar task designs, and also being in line with a previous study investigating the neural bases of face and body perception using a functional region-of-interest approach with a sample size of N = 35 participants reporting F-values above 40 for all repeated measures analyses (Julian et al. 2012 Neuroimage).
Data exclusions	No data were excluded from the analysis.
Replication	We replicated previous findings in human neuroimaging. By adding bodies as stimuli for the first time, and thus controlling for animacy, our findings expand those from earlier investigations on face perception in dogs, and suggest that previously identified face-sensitive areas respond more generally to animate entities. This is the first study disentangling the neural bases of animate vs. inanimate and face vs. body perception in dog neuroimaging and has not been replicated yet.
Randomization	We used the same task for dogs and humans.
Blinding	Blinding was not relevant in this study design, since we did not have different experimental groups.

Reporting for specific materials, systems and methods

We require information from authors about some types of materials, experimental systems and methods used in many studies. Here, indicate whether each material, system or method listed is relevant to your study. If you are not sure if a list item applies to your research, read the appropriate section before selecting a response.

Materials & experimental systems

n/a	Involved in the study
x	Antibodies
x	Eukaryotic cell lines
x	Palaeontology and archaeology
	x Animals and other organisms
x	Clinical data
x	Dual use research of concern

Methods

n/a	Involved in the study
x	ChIP-seq
x	Flow cytometry
	x MRI-based neuroimaging

Animals and other research organisms

Policy information about studies involving animals; ARRIVE guidelines recommended for reporting animal research, and Sex and Gender in Research

Laboratory animals	The study did not involve laboratory animals.
Wild animals	The study did not involve wild animals. All dogs participating were pet dogs living in family homes. We collected data from N = 15 pet dogs (age range: 4-11 years). They all received extensive training prior to study participating to habituate to the scanner environment (Karl et al. 2020 Behav. Res. Methods) and were fully awake and unrestrained throughout data collection.
Reporting on sex	The majority of the dogs were female (11 out 15 dogs). Sex differences were not considered in the analysis since be investigated basic cognitive mechanisms (i.e., visual perception of faces and bodies).
Field-collected samples	We did not collect any data in the field.
Ethics oversight	Dog data collection was approved by the institutional ethics and animal welfare commission in accordance with Good Scientific Practice (GSP) guidelines and national legislation at the University of Veterinary Medicine Vienna (ETK-06/06/2017), based on a pilot study conducted at the University of Vienna.

Note that full information on the approval of the study protocol must also be provided in the manuscript.

Magnetic resonance imaging

Experimental design

Design type	block-design (task-based)
Design specifications	two 5 min runs, humans: 1 session (humans), dogs: on average 3 sessions, 6 trials times 6 conditions, block-length: 12 s (5 images per block), jittered visual baseline between 3-7 seconds
Behavioral performance measures	passive viewing paradigm

Acquisition

Imaging type(s)	functional
Field strength	3 Tesla
Sequence & imaging parameters	dogs: GE, EPI, MB-EPI factor = 2, TR/TE = 1000/38 ms, voxel size = 1.5 x 1.5 x 2 mm^3, FoV = 144 × 144 × 58 mm3, flip angle = 61°, humans: dogs: GE, EPI, MB-EPI factor = 4, TR/TE = 2100/3.13 ms, voxel size = 2 mm isotropic, FoV = 230 × 230 × 165 mm3, flip angle = 66°
Area of acquisition	whole-brain

Diffusion MRI ☐ Used ☒ Not used

Preprocessing

Preprocessing software	SPM12 (https://www.fil.ion.ucl.ac.uk/spm/software/spm12/)

Normalization	non-linear transformation of structural and EPI images to species-specific templates
Normalization template	human data: MNI space; dog data: breed-averaged template (Nitzsche et al 2019 Neuroimage)
Noise and artifact removal	six realignment parameters in GLM and additional motion scrubbing (see below)
Volume censoring	We calculated individual scan-to-scan motion (framewise displacement, FD) and added motion regressors in GLM for each scan exceeding the a priori set FD threshold of .5 mm (i.e., motion scrubbing; Power et al. 2012,2014 Neuroimage) to account for both translational and rotational displacements. For the dog participants, we removed on average 8% of the scans from each run (run 1: mean FD = .23 mm, 90th percentile = .36 mm; run 2: mean FD = .24 mm, 90th percentile = .38 mm) and 1% of the scans from each run of the human participants (run 1: mean FD = .17 mm, 90th percentile = .22 mm; run 2: mean FD = .18 mm, 90th percentile = .21 mm).

Statistical modeling & inference

Model type and settings	mass univariate: functional region-of-interest (fROI; repeated measures analyses of variance, ANOVAs) and whole-brain (ANOVA using the flexible factorial framework in SPM12 with same levels as fROI analysis); one-sample t-test of the contrast all stimuli > baseline to determine visual-responsive areas) analysis and whole-brain multivariate RSA (permutation-based paired t-test on group-level).
Effect(s) tested	mass univariate: BOLD-signal changes (i.e., activation levels) between conditions-of-interest using ANOVAs. Levels for main analysis: faces, bodies, inanimate objects; all levels > scrambled controls. For species analysis, two factors: species (dog, human), image category (face, body). RSA: comparison of pattern similarities between trials of (a) faces vs. inanimate objects (i.e., [dog faces × human faces] vs. inanimate objects), (b) bodies vs. inanimate objects (i.e., [dog bodies × human bodies] vs. inanimate objects), (c) animate vs. inanimate images (i.e., [dog faces × human faces x dog bodies × human bodies] vs. inanimate objects), (d) faces vs. bodies (i.e., [dog faces × human faces] vs. [dog bodies × human bodies]), and within the face and body categories: images of conspecifics vs. heterospecifics (i.e., (e) dog faces vs. human faces; (f) dog bodies vs. human bodies).

Specify type of analysis: ☐ Whole brain ☐ ROI-based ☒ Both

Anatomical location(s)	We used a functional region-of-interest (fROI) analysis which relied on splitting the data into two independent data sets: (a) a localizer data set (first task run) to define individual potential face- or body-sensitive areas in visual-responsive brain regions and (b) a test data set (second task run) to extract activation levels from these regions. Anatomical masks from Czeibert et al. (2019, Biol. Futur. normalized to the breed-averaged template space from Nitzsche et al. 2019 Neuroimage) of the gyri containing visual-responsive activation (all stimuli > baseline, task run 1) served as anatomical search spaces for the fROI analysis of the dogs. For humans we used parcels derived from a previous study (Julian et al. 2012 Neuroimage). In an additional control analysis for the human data, we used anatomical masks of the fusiform gyrus (Shattuk et al. 2008 Neuroimage) and calcarine sulcus including the surrounding cortex (Rolls et al. 2015 Neuroimage) to serve as search spaces.

Statistic type for inference (See Eklund et al. 2016)	Cluster-defining threshold of p < .005/.001 (dogs/humans) and a cluster probability of p < .05 family-wise error (FWE) corrected for multiple comparisons. Cluster extent (i.e., minimum spatial extent to be labelled significant) was calculated using the SPM extension "CorrClusTh.m" (https://warwick.ac.uk/fac/sci/statistics/staff/academic-research/nichols/scripts/spm/spm8/corrclusth.m)
Correction	FWE (whole-brain univariate and RSA), FDR (functional region-of-interest analysis)

Models & analysis

n/a	Involved in the study
☒	☐ Functional and/or effective connectivity
☒	☐ Graph analysis
☒	☐ Multivariate modeling or predictive analysis
